# Green synthesis of silver nanoparticle using pollen extract from *Tetragonisca angustula* a stingless bee

**DOI:** 10.1186/s11671-024-04038-0

**Published:** 2024-05-27

**Authors:** Ana Carolina Costa Santos, Gabriela Carvalho Batista, Rafaela Cavalcante Cerqueira, Mariana Gonçalves Lisboa, Joberth Lee Correa, Tamiris Sabrina Rodrigues, Murillo Néia Thomaz da Silva, Vinícius Prado Bittar, Serena Mares Malta, Natalia Carine Lima dos Santos, Foued Salmen Espindola, Ana Maria Bonetti, Carlos Ueira-Vieira

**Affiliations:** 1 Laboratory of Genetics, Institute of Biotechnology, Federal University of Uberlândia, Uberlandia, Brazil; 2 Laboratory of Nanobiotechnology, Institute of Biotechnology, Federal University of Uberlândia, Uberlandia, Brazil; 3 Laboratory of Biochemistry and Molecular Biology, Institute of Biotechnology, Federal University of Uberlandia, Uberlandia, Brazil

**Keywords:** Stingless bees, Nanoparticles, Sustainable, Antimicrobial

## Abstract

This study explores the green synthesis of silver nanoparticles (AgNPs) using a methanolic extract of fermented pollen from *Tetragonisca angustula*, a species of stingless bees. The AgNPs exhibit spherical morphology, low charge values, and suspension stability, with their unique composition attributed to elements from the pollen extract. Antioxidant assays show comparable activity between the pollen extract and AgNPs, emphasizing the retention of antioxidant effects. The synthesized AgNPs demonstrate antimicrobial activity against multidrug-resistant bacteria, highlighting their potential in combating bacterial resistance. The AgNPs exhibit no toxic effects on *Drosophila melanogaster* and even enhance the hatching rate of eggs. The study underscores the innovative use of stingless bee pollen extract in green synthesis, offering insights into the varied applications of AgNPs in biomedicine.

## Introduction

Nanoparticles synthesized from silver nitrate have been widely used for therapeutic purposes due to their physical, chemical, and biological properties [1, 2]. Nanoparticles are utilized in diagnostics, therapies, and pharmaceutical production owing to their minute dimensions [3, 4]. The synthesis of these nanoparticles can occur through two main approaches: Top-Down and Bottom-Up. In the Top-Down method, such as laser ablation and spraying, materials are directly shaped and utilize physical techniques such as milling, cutting, and shaping with tools [5–7]. In the Bottom-Up method, which involves chemical or biological processes, nanoparticles are built from smaller components, allowing atoms or molecules to join to form larger particles [7–9].

Chemical synthesis, using inorganic or organic reducing agents, is recognized for its high efficiency compared to other techniques, although it may result in the production of toxic and environmentally harmful residues [4, 10]. Biological synthesis or green synthesis involves the reduction of silver ions into AgNPs, facilitated by a solvent, an environmentally friendly reducing agent, and a non-toxic stabilizing agent [11]. These methods can involve the use of plant extracts, bacteria, fungi, and other biomaterials [8, 12–16]. Green synthesis results in the production of more stable nanoparticles with increased biocompatibility, minimizing environmental residues by utilizing sustainable and natural compounds in the synthesis process, thereby reducing the generation of harmful by-products [17, 18]. On the other hand, silver nanoparticles (AgNPs) generated through physical and chemical synthesis methods typically yield nanoparticles with consistent size and morphology but incur higher production costs and pose potential toxicity risks to animals and humans [6, 19].

The green production of AgNPs using reducing agents with antioxidant activity has gained attention due to the ability of these compounds to stabilize the nanoparticles and enhance their effects [20, 21]. For example, plant extracts rich in polyphenols, flavonoids, or enzymes have been instrumental in mediating the reduction of silver ions into AgNPs [12, 22]. Products from stingless bees constitute a source of biomolecules due to their unique composition, high nutritional value, and potential for biotechnological use [23, 24]. The honey, geopropolis, and pollen from these bees have anticancer, antioxidant, anti-inflammatory, antimicrobial, among other actions [25–28]. Stingless bee pollen includes pollen grains collected by bees from diverse botanical sources, serving as a potent reservoir of antioxidants due to the presence of flavonoids and phenolic compounds. Additionally, it can function as a reducing agent in the synthesis of silver nanoparticles [24, 29, 30].

Here, we employ a methanolic extract of fermented pollen collected from *Tetragonisca angustula* as a reducing agent in the green synthesis of silver nanoparticles (AgNPs). Furthermore, we evaluated the antioxidant and antimicrobial activities of the generated nanoparticles and their cytotoxic potential in *Drosophila melanogaster*.

## Results

### Nanoparticles synthesis

**Fig. 1:**
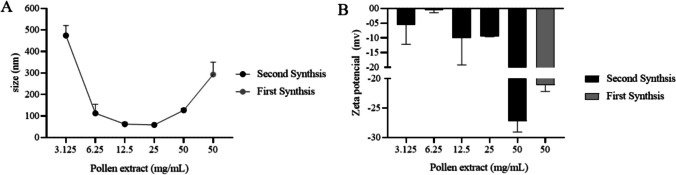
Dynamic light scattering (**A**) and zeta potential analysis (**B**) of AgNPs produced with pollen extract

The green nanoparticles synthesized using 500 mg of pollen extract from *T. angustula* and 20 mg of AgNO_3_ yielded of 70 mg of AgNPs with an average size of 293.28 nm (Fig. 1A). The zeta potential measured at -21.13 shows a negative charge on these nanoparticles (Fig. 1B).

A second synthesis run was conducted, using varying quantities of pollen extract from *T. angustula* and doubling the amount of AgNO_3_ used in the initial synthesis. Specifically, the use of 125 and 250 mg of pollen extract resulted in the production of smaller AgNPs, measuring 62.17 nm and 58.55 nm, respectively. However, when trying to dry the AgNPs in microtubes for weight measurement, they adhered to the inner surfaces of the tubes, rendering it impossible to accurately determine the yield, through this technique. Consequently, all subsequent analyses were conducted solely with AgNPs from the first synthesis.

### UV–Vis spectra and Fourier transform infrared spectroscopy (FTIR) analysis

**Fig. 2:**
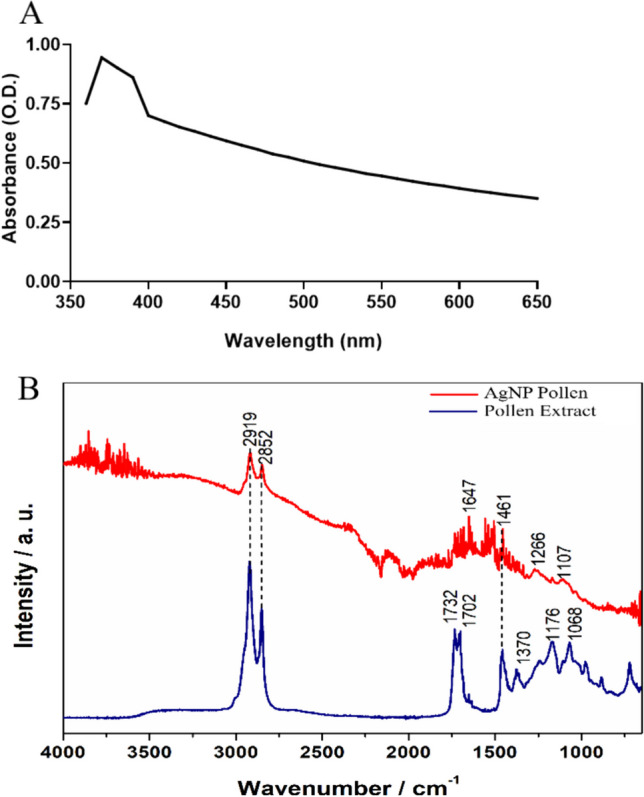
Analysis of UV–visible (**A**) spectrum and FT-IR (**B**) of pollen extract, and AgNPs produced with 50 mg/ml pollen extract

Within the UV–visible spectrum, a prominent peak appeared in the 230–300 nm range, concomitant with a significant decline near 350 nm (Fig. 2A). To obtain local-scale organizational structural information, short-range absorption spectroscopy measurements in the infrared region were conducted (Fig. 2B).

The obtained Infra-Red spectra display vibrational modes associated with the presence of characteristic organic functional groups found in the biological materials used in the synthesis process. Comparing the vibrations in the spectra of different materials, the presence of bands at similar positions is clear.

### SEM-EDX analysis

**Fig. 3:**
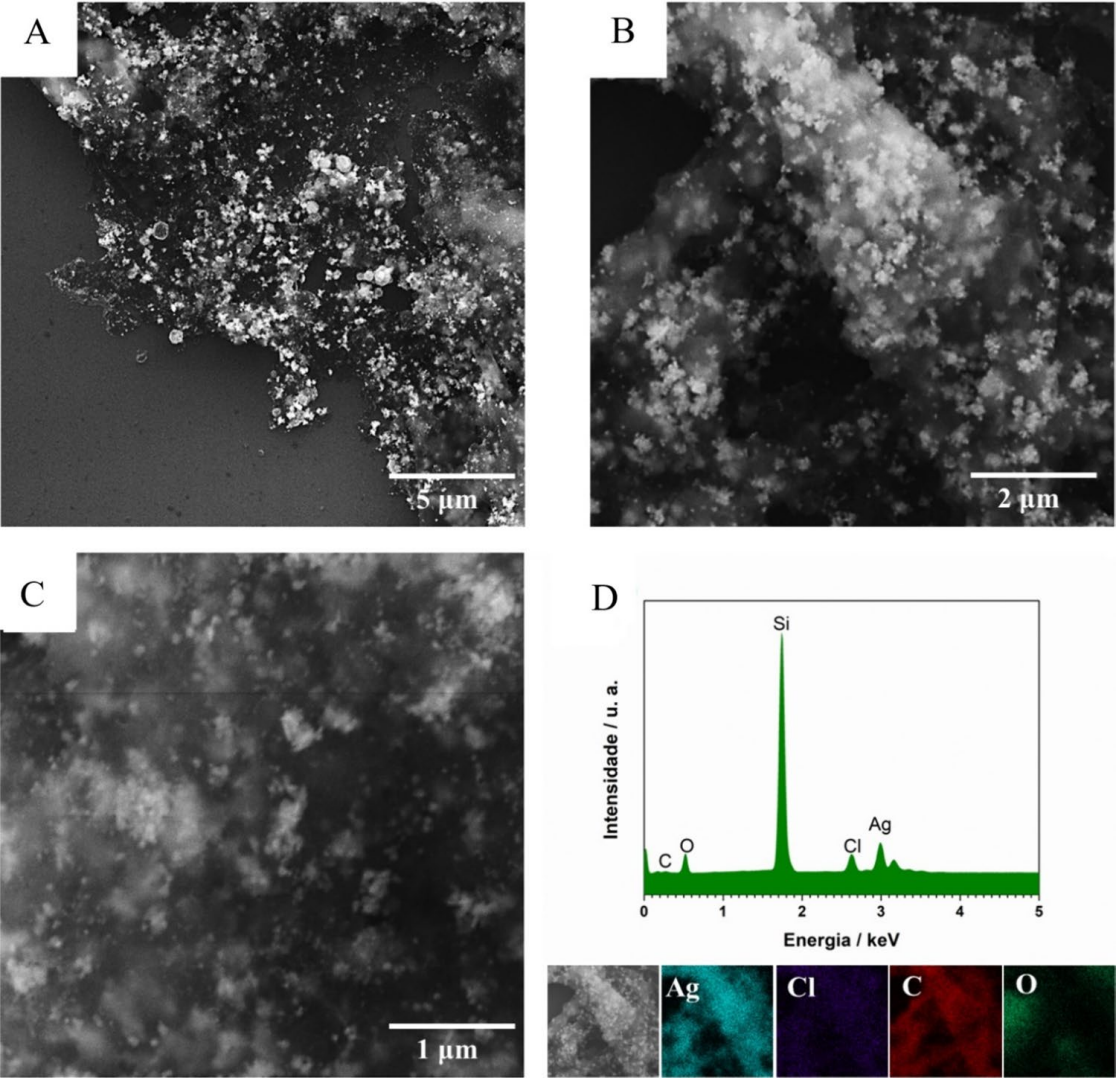
**A**–**C** scanning electron microscopy images at different magnifications, and **D** EDX spectrum and mapping of Pollen AgNPs

The scanning electron microscopy technique was employed to investigate the size and morphology of the synthesized materials. Figure 3A–C present SEM images. The images obtained for sample Pollen AgNPs exhibit a spherical morphology with average diameters ranging from 10 to 50 nm. The EDX spectra and mappings presented for sample Pollen AgNPs (Fig. 3D) indicate the presence of silver, suggesting the nanoparticle are really synthetized. The presence of chlorine, carbon, and oxygen elements implies that these elements are deposited on the surface of the nanoparticles, originating from the pollen extract.

### Antioxidant activity of green Ag NPs

**Fig. 4:**
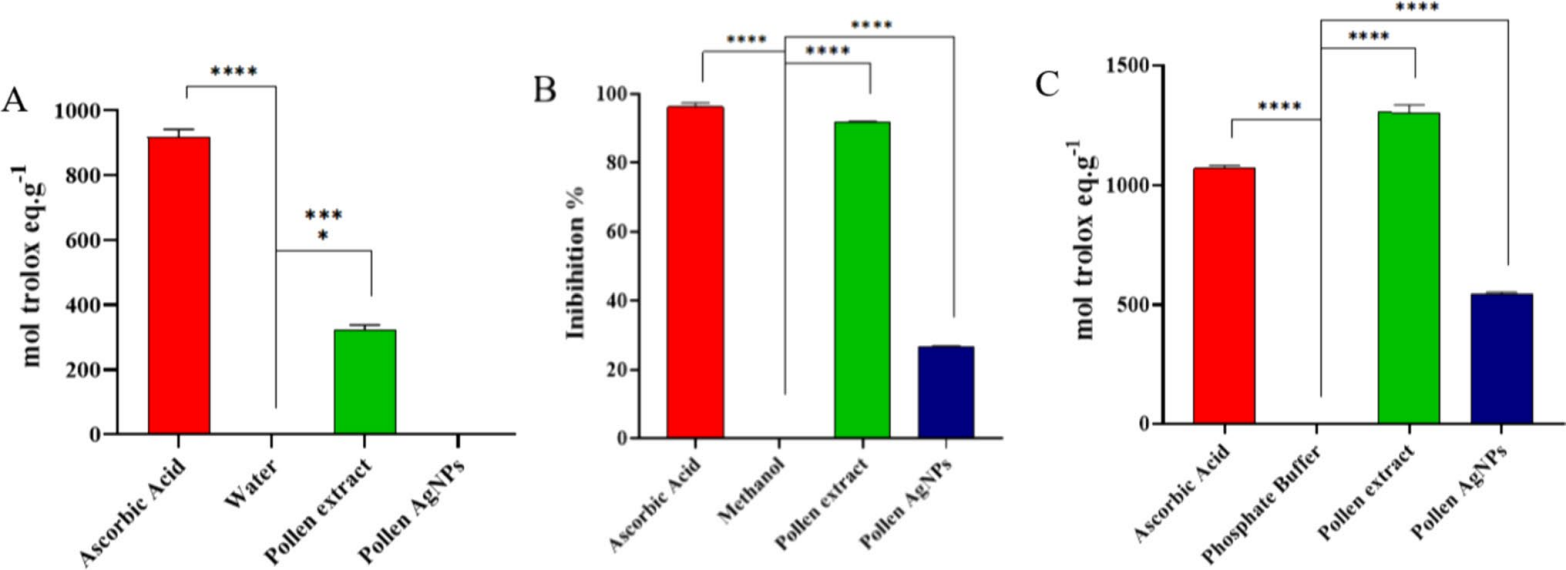
Antioxidant activity tests FRAP (**A**), DPPH (**B**) and ORAC (**C**) of Pollen AgNPs

In Ferric Reducing Antioxidant Power (FRAP), Oxygen Radical Absorbance Capacity (ORAC), and 2,2-diphenyl-1-picrylhydrazyl (DPPH assays) (Fig. 4), the pollen extract exhibited antioxidant activity. In DPPH (Fig. 4B) and ORAC (Fig. 4C) tests, it showed antioxidant activity like the control, ascorbic acid, which is known for its antioxidant properties. Nanoparticles synthesized with silver nitrate and pollen extract showed antioxidant activity in DPPH and ORAC tests, but not in iron reduction (FRAP). In contrast, the pollen extract exhibited antioxidant activity in all three assays (FRAP, ORAC, and DPPH). While the nanoparticles effectively reduced the DPPH free radical and inhibited the oxidation of oxygen free radicals, they did not demonstrate the same ability to reduce iron ions as the pollen extract, which appears to contain substances that also reduce iron ions in addition to reducing DPPH and oxygen free radicals.

### Antimicrobial assay

The antimicrobial effects of pollen AgNPs were evaluated in two strains of multidrug-resistant bacteria. In the disk diffusion assay, the nanoparticle produced with pollen extract exhibited inhibition effects against *E. coli* and *S. aureus*. However, when using only the pollen extract, no antimicrobial effects were observed (Table 1), indicating the potential of the produced nanoparticles for use against both bacteria multirresistent.

**Table 1:** Result of hallo formation in diffusion disk assay

	Inhibition zone (mm mean ± SD)
*E. coli*	
Pollen extract	0
AgNPs pollen	11.59 ± 0.45
Pollen extract	0
*S. aureus*	
AgNPs pollen	8.33 ± 0.98

**Fig. 5:**
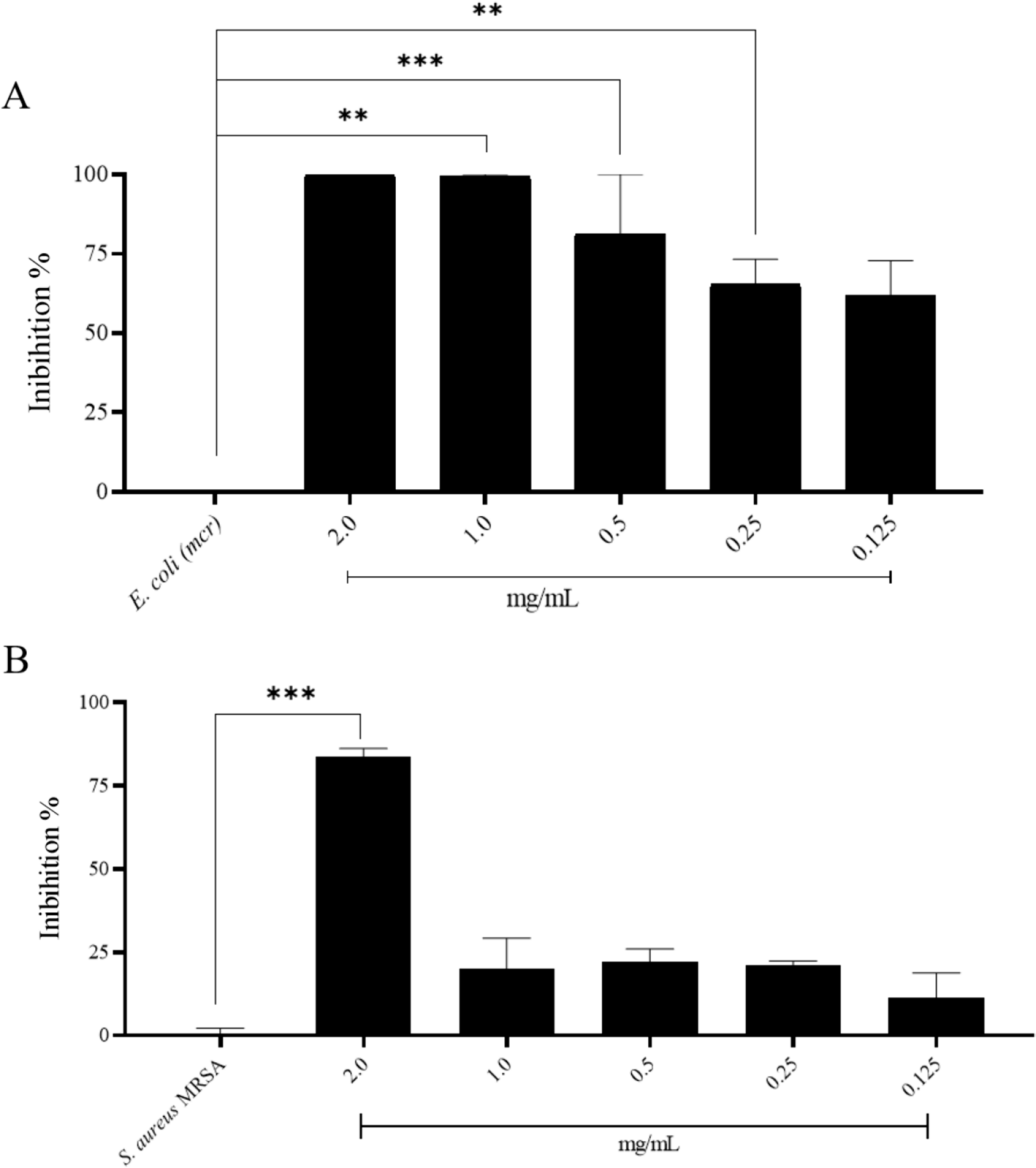
Results of the antimicrobial action in MIC of AgNPs synthesized. **A** Inhibition results in *E. coli* *mcr*; **B** Inhibition results in *S. aureus* MRSA

The minimum inhibitory concentration (MIC) was found to be 2.0 mg/mL for *Staphylococcus aureus* MRSA and 0.5 mg/mL for *E. coli mcr*. These findings highlight the compound's heightened effectiveness against gram-negative bacterial (Fig. 5A) strains compared to their gram-positive counterparts (Fig. 5B).

### Toxicity evaluation in *Drosophila melanogaster*

**Fig. 6:**
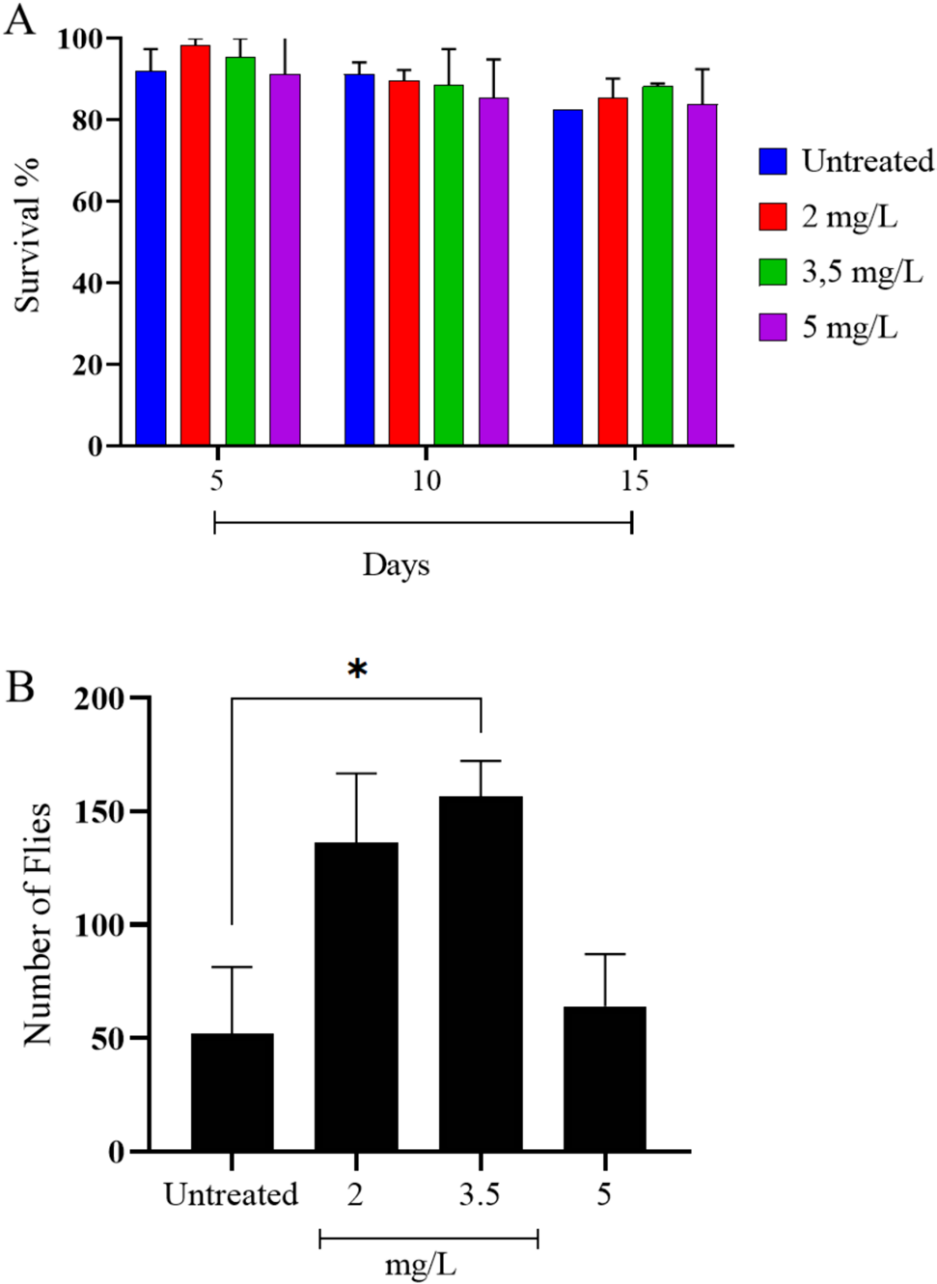
Toxicity evaluation of the AgNPs in *Drosophila melanogaster.* **A** Toxicity assay in *D. melanogaster*; **B** Effects in eclosion number of in *D. melanogaster*

The nanoparticles synthesized with silver nitrate showed no toxic effects on the Canton-S fly strain over a 15-day treatment period (Fig. 6A). Furthermore, it did not affect the viability of their eggs. Treatments with 3.5 mg/mL of AgNPs exhibited an increase in the hatching rate of Drosophila (Fig. 6B).

## Discussion

The *Tetragonisca angustula* is a small stingless bee widely spread into forests and urban areas in Central and South America. These stingless bees play a pivotal role in the pollination of diverse plant species, thereby making a significant contribution to biodiversity and food production [31–33]. Their pollen possesses exceptional value due to its nutritional composition and medicinal properties [31, 32]. Recently our research group has analyzed the metabolites composition of its pollen, and we showed the methanolic pollen extract has considerable antioxidant potential, and is composed of flavonoids, tannins, and polyphenols [34]. The compounds have gained great visibility for their proven antioxidant, antimicrobial, anticancer action, among other health benefits [26, 34, 35].

Here we show that the compounds present in the methanolic pollen extract of *T. angustula* were able as a reducing agent for use in the green synthesis of AgNP. Different concentrations of methanolic pollen extract generated differences in size and charge. Increasing the concentration of pollen extract increased the absorbance intensity of the nanoparticles and their stability. The biomolecules present in the pollen extract may be acting as reducing agents, preventing aggregation, and maintaining the stability of the nanoparticles [36–38]. Conversely, with an increase in the amount of AgNO_3_ and a reduction in the amount of extract, the nanoparticles decreased in size and zeta potential [7, 38].

The AgNPs generated with of methanolic pollen extract exhibit low charge values (below − 20 mV), indicating robust physical stability in the suspension. This stability is attributed to the electrostatic repulsion among individual nanoparticles [39]. The zeta potential of nanoparticles synthesized with pollen extract suggests that the flavonoids, terpenes, and tannins present in the pollen extract function as stabilizing agents, keeping them stable in solution and inhibiting aggregation [40].

Green-synthesized AgNPs exhibit variations in both shape and size, with spherical, triangular, and hexagonal forms being the most prevalent [41]. Here, we observed that AgNPs from pollen extract are spherical. However, it is important to emphasize that *T. angustula* has the capability to collect various types of pollen during different seasons due to the flowering patterns of surrounding plants at the collection site [31]. Therefore, various pollen extracts gathered from the same beehive may yield nanoparticles of different sizes and shapes.

Silver nanoparticles from chemical synthesis are recognized for displaying a UV–visible absorption peak within the 400–500 nm range, attributed to surface plasmon resonance [42]. Nevertheless, in the context of green synthesis involving complex mixtures, various compounds can attach to the silver atom, leading to the formation of stable nanoparticles. These compounds have the potential to modify the absorption spectrum. Even in chemical synthesis is possible to visualize peaks raging 330–360 nm [10].

The presence of spectral peaks at 2919, 2852, and 1455 cm^−1^ in FTIR spectroscopy analysis of AgNPs from pollen methanolic extract can be attributed to the stretching vibration of aliphatic C-H hydrocarbon chains and N–H bending vibration [43, 44]. The spectral vibration in the range 1650–1621 cm^−1^ can be attributed to the stretching of amides (N–H) in addition to the peptide bond and C=C stretching, which may be involved in nanoparticle stabilization by proteins [45]. The bands centered at 1266, and 1107 cm^−1^ correspond to the stretching of N–H bonds in amines, stretching of C–O bonds, and the vibration of C–N bonds in protein amines, respectively [46, 47]. The bands between 1500 and 800 cm^−1^ correspond to the characteristic signal of phenolic compounds and are due to the vibration of C–C and C–O in phenolic and flavonoid compounds [48]

Nanoparticles synthetized using plant extracts retain some of their antioxidant effects, as they may be incorporating some of the compounds responsible for the effect [13, 20, 21, 49]. In this study, the pollen extract from *T. angustula* exhibited an antioxidant effect comparable to that of ascorbic acid, possibly due to the presence of phenolic extracts and flavonoids in the pollen, which may be linked to the antioxidant effect [29, 30]. These compounds may be associated with the produced nanoparticles, as they exhibited antioxidant effects in DPPH and ORAC assays.

Antimicrobial resistance poses a significant global public health challenge. Recently, silver nanoparticles produced through chemical synthesis have been gaining prominence in research demonstrating their effectiveness against various bacteria resistant to commercial antibiotics [50]. In this study, our findings demonstrate the efficacy of silver nanoparticles (AgNPs) derived from methanolic extract of pollen against both *Escherichia coli mcr* and *Staphylococcus aureus* MRSA. Notably, this research marks the pioneering use of pollen methanolic extract from stingless bees. Given the substantial variability in the minimum inhibitory concentration (MIC) of AgNPs, especially in the context of green synthesis, the absence of a standardized parameter for comparison underscores the need for further investigation in this novel application.

The *Escherichia coli*, the most prevalent gram-negative microorganism, has evolved various resistance mechanisms, complicating the treatment of infections. Serious bacterial infections characterized by resistance to a broad spectrum of antibiotics, including fluoroquinolones, aminoglycosides, carbapenem, and ß-lactams, have witnessed a concerning rise in recent years. The scarcity of new antimicrobial agents has prompted a reevaluation of colistin [51, 52]. This drug has now regained clinical significance as a last-resort option for treating infections caused by Gram-negative bacteria [53].

The *Staphylococcus aureus* methicillin-resistant strain, commonly known as MRSA, denotes a gram-positive bacterial variant displaying resistance to methicillin and other beta-lactam antibiotics. Its resistance mechanism is attributed to the acquisition of the *mecA* gene, encoding an altered penicillin-binding protein [54, 55]. MRSA infections present formidable challenges in clinical settings and communities due to their limited therapeutic options [56]. Addressing MRSA necessitates a comprehensive approach encompassing meticulous infection control strategies, vigilant surveillance, and the judicious use of alternative antibiotics [57].

The specific minimum inhibitory concentration (MIC) of silver nanoparticles (AgNPs) against bacteria may vary depending on the size, shape, and synthesis method of the nanoparticles, as well as the testing conditions [58]. As an example, AgNPs green-synthesized using extracts from Pu-erh tea leaves demonstrated variations in MIC values within the microgram per milliliter range for different bacterial strains. These strains were sourced from the American Type Culture Collection (Rockville, MD, United States) and did not exhibit a multidrug-resistant phenotype [59]. In our investigation, we opted to exclusively examine the impact on multidrug-resistant bacteria, given the substantial health challenges they present.

Silver nanoparticles synthesized through chemical synthesis have shown adverse effects in studies involving fruit flies and other organisms, reducing lifespan and fecundity [60]. The green synthesis shows a tendency to exhibit lower toxicity compared to conventional chemical and physical synthesis methods [8, 17, 41]. The concentrations of silver nanoparticles used did not demonstrate toxicity in adult *Drosophila melanogaster* or embryos; however. Pollen extract, in its isolated form, is known to contain compounds that promote the survival of *Drosophila* [34]. Our results suggest that some of these compounds have been incorporated into the nanoparticles, thereby preserving the therapeutic properties inherent in pollen extract.

## Conclusion

This study concluded that pollen extract exhibits antioxidant action, and the nanoparticle produced using it maintains antioxidant properties while preserving nanoparticle stability. The AgNPs from *T. angustula* pollen extract exhibits antimicrobial properties against both *E. coli* and *S. aureus,* multidrug-resistant bacteria and showed no toxicity against *D. melanogaster*. This research highlights the viability of utilizing extract from stingless bee pollen as a source of substances with the potential for green synthesis of nano compounds and nanomaterial, highlighting significant innovation in the realm of healthcare.

## Material and methods

### Pollen extract preparation

Pollen pots from two colonies of *Tetragonisca angustula* stingless bees located in an urban Meliponary in Uberlândia city, Minas Gerais State, Brazil, in June 2020. The collected pollen was then transferred to a beaker, and a mixture with 581 mL of absolute methanol in a 1:7 ratio (m/v), followed by 30 min of stirring. Subsequently, the blend was left undisturbed in the absence of light at room temperature for 88 h. The resulting liquid underwent filtration, followed by processing using a rotary evaporator. The obtained extract underwent lyophilization and freezing, yielding 11 g of crude extract.

### First snthesis

In the initial step of the green synthesis process, 500 mg of methanolic pollen extract was combined with 10 mL of a 0.01% Tween 80 solution (Sigma), which had been diluted in distilled water. Subsequently, 20 mg of AgNO_3_ (Sigma) was introduced into the solution, followed by the addition of 0.5 mL of NH_4_OH [61]. The mixture was manually stirred until a noticeable change in color occurred. The solution underwent microwaving for 30 s, divided into three intervals of 10 s each. Following this procedure, the nanoparticles were subjected to three washes, each involving 5 mL of distilled water, and were subsequently centrifuged at 10,000 g for 10 min. The resulting pellet was then resuspended in 1 mL of ultrapure water (Milli-Q).

### Second nanoparticles synthesis

In the pursuit of optimizing the efficiency in silver nanoparticle synthesis, concomitantly evaluating the potential impact of pollen extract quantity on the resultant yield, five solutions were prepared, each containing 500, 250, 125, 62.5, and 31.25 mg of methanolic pollen extract were dissolved with 10 mL of a 0.01% Tween 80 solution. Subsequently, 40 mg of AgNO_3_ was introduced and completely dissolved into each solution, followed by the addition of 0.5 mL of NH_4_OH. The subsequent steps followed the same process as the initial synthesis. The mixtures were manually stirred until a noticeable change in color occurred. The solutions were then subjected to microwaving for 30 s, divided into three intervals of 10 s each. The nanoparticles were washed as described in the first synthesis.

### UV–Vis spectra analysis

After the green synthesis, the AgNPs were characterized. UV–visible spectroscopy analyses were performed using an EnSpire Multimode Plate Reader (PerkinElmer). Ultrapure water was used as blank to adjust the baseline.

### Infrared spectroscopy

The spectra in the infrared region were obtained using an Agilent Cary 630 FTIR spectrophotometer, in the range between 4000 and 650 cm^−1^. Sample analyses were performed in the solid state, using the Attenuated Total Reflectance (ATR) accessory with a diamond crystal.

### Size measurement and zeta potential by dynamic light scattering (DLS)

The DLS analyses to verify particle size and zeta potential were conducted using the Particle Analyzer Litesizer 500 (Anton Paar), according to manufacture. Ultrapure water was used to dilute green AgNPs at 1:1000.

### Scanning *electron* microscopy (SEM) and energy dispersive X-ray spectroscopy

For obtaining the SEM images, a TESCAN Vega 3 scanning electron microscope operated at 20 kV was used, along with a secondary electron detector and an EDX detector (Oxford Instruments, Bucks, England). A voltage of 20 kV was applied for acquiring the EDX spectra and mapping.

### Antioxidant assay

The antioxidant potential of the pollen extract and nanoparticles was evaluated by FRAP (ferric reducing antioxidant power), ORAC (Oxygen radical absorbance capacity) and DPPH (2,2-diphenyl-1-picrylhydrazyl) assays [49].

For the FRAP assay, 10 μL the samples were combined with 250 μL of FRAP reagent, consisting of 10 × sodium acetate 0.3 M, TPTZ (2,3,5-triphenyltetrazolium chloride) 10 mM, and 1 volume of ferric chloride 20 mM. Additionally, 25 µl of water were added, and the mixture was incubated at 37 °C for a duration of 6 min. The positive control included ascorbic acid in concentration of 0.25 mg/ml, while the negative control was water. Following the incubation period, the absorbance was measured at 593 nm using a plate reader. The determination of the antioxidant capacity for each sample involved the creation of an analytical curve utilizing Trolox (6-hydroxy-2,5,7,8-tetramethylchroman-2-carboxylic acid)[49, 62].

For the DPPH assay, 75 μL of samples and 225 μL of 2,2-diphenyl-1-picrylhydrazyl (DPPH) at 0.06 mM were added in well. The positive control included ascorbic acid in concentration of 0.25 mg/ml, while the negative control was methanol. The plate was incubated at 30 °C for 30 min in the dark, and the absorbances of the samples were read using a spectrophotometer at a wavelength of 517 nm. Antioxidant activity is obtained depending on the percentage of DPPH neutralized, using the formula: DPPH (%) = [(X control − X sample)/ (X control − X blank)] 100 [29, 49].

In the ORAC assay, samples underwent incubation with 0.085 mM fluorescein and 153 mM 2,2′-azobis(2-amidinopropane) dihydrochloride (AAPH), both reagents diluted in 75 mM phosphate buffer (pH 7.4). Fluorescence intensity was measured at 485 nm excitation and 528 nm emission over a 90-min duration at 37 °C using a microplate reader. The negative control comprised phosphate buffer, while ascorbic acid functioned as the positive control. To evaluate antioxidant capacity, an analytical standard curve of trolox was generated, and the results were quantified and expressed as μmol of trolox per gram (eq. g − 1) [49].

### Antimicrobial activity of green Ag NPs

#### Disc diffusion susceptibility

The potential of pollen extract and green AgNps of inhibit bacterial growth were evaluated using MIC, against multidrug-resistant *E. coli* (*mcr*) and *S. aureus* (MRSA) bacteria, provided by the Molecular Microbiology Laboratory of the Federal University of Uberlândia, all bacteria were genotyped by PCR and phenotype by growing on BHI agar supplemented with ampicillin and polymyxin, respectively. The antimicrobial effect was determined using the modified Kirby–Bauer disk diffusion test [63, 64] Following the streaking of bacteria on the agar plate at a concentration of 0.5 on the McFarland scale, 15 µl (50 mg/mL) of pollen extract and the produced nanoparticles (50 mg/ml) were applied to an antimicrobial susceptibility testing disc, in triplicate. The discs were then incubated at 37 °C for a duration of 24 h. Post-incubation, the presence of inhibition zones was assessed, and the diameters of the formed halos were measured using calipers.

#### Minimum inhibition concentration

Green AgNps were evaluated for their ability to inhibit the growth of multidrug-resistant E. coli *(mcr)* The antimicrobial effect was determined using plate microdilution method. The pathogenic bacteria used were obtained from culture collections or isolated from clinical samples, provided by the Molecular Microbiology Laboratory (MICROMOL) of the Federal University of Uberlândia. For the assay, the bacterial suspension was prepared in LB broth at 37 °C ± 1 for 24 h and the bacterial concentration was adjusted to 0.5 McFarland standard. To determine the minimum inhibitory concentration (MIC) of the nanoparticles with antimicrobial effect in the disk diffusion test, dilutions ranging from 2 to 0.125 mg/mL were performed. One hundred microliters of bacterial suspension were transferred to a 96-well plate containing 100 μL of nanoparticles to evaluate whether it would reduce bacterial growth. Wells containing 200 μL of bacteria and LB were used as positive and negative controls for bacterial growth, respectively. The plate was incubated for 24 h at 37 °C  ± 0.5 and bacterial growth was evaluated in a microtiter plate reader at 595 nm at 24 h [25].

#### Toxicity evaluation in *Drosophila melanogaster*

*Drosophila melanogaster* wild-type (Canton-S) strain flies, aged between 0 and 2 days post-eclosion, (d.p.e) were sorted into groups of 20 individuals with a 1:1 male-to-female ratio and housed in a chamber at 25 °C under a 12/12-h light/dark cycle. For treatment administration, 0.5 g of potato mash enriched with 75% instant potato mash, 15% yeast extract, 9.3% glucose, and 0.07% nipagin was hydrated with nanoparticle solutions at concentrations of 2, 3.5, and 5 mg/mL [60, 62]. A control group was included using distilled H2O. Treatment media were replaced every two days, and the number of deceased individuals was recorded at each replacement. The total exposure time of the flies to nanoparticle-containing diet was 15 days.

Vials containing eggs from the toxicity experiment, after being emptied for medium exchange, were kept in an incubator for 13 days, and the hatched individuals were counted to measure the effects of exposition of AgNP in the rate of eclosion.

## Data Availability

All data supporting the findings of this study are available within the paper.
